# Deformable appearance pyramids for anatomy representation, landmark detection and pathology classification

**DOI:** 10.1007/s11548-017-1622-5

**Published:** 2017-06-03

**Authors:** Qiang Zhang, Abhir Bhalerao, Charles Hutchinson

**Affiliations:** 1 Department of Computer Science, University of Warwick, Coventry, UK; 2 University Hospitals Coventry and Warwickshire, Coventry, UK

**Keywords:** Deformable part models, Deformable appearance pyramids, Landmark detection, Classification

## Abstract

**Purpose:**

Representation of anatomy appearance is one of the key problems in medical image analysis. An appearance model represents the anatomies with parametric forms, which are then vectorised for prior learning, segmentation and classification tasks.

**Methods:**

We propose a part-based parametric appearance model we refer to as a deformable appearance pyramid (DAP). The parts are delineated by multi-scale local feature pyramids extracted from an image pyramid. Each anatomy is represented by an appearance pyramid, with the variability within a population approximated by local translations of the multi-scale parts and linear appearance variations in the assembly of the parts. We introduce DAPs built on two types of image pyramids, namely Gaussian and wavelet pyramids, and present two approaches to model the prior and fit the model, one explicitly using a subspace Lucas–Kanade algorithm and the other implicitly using the supervised descent method (SDM).

**Results:**

We validate the performance of the DAP instances with difference configurations on the problem of lumbar spinal stenosis for localising the landmarks and classifying the pathologies. We also compare them with classic methods such as active shape models, active appearance models and constrained local models. Experimental results show that the DAP built on wavelet pyramids and fitted with SDM gives the best results in both landmark localisation and classification.

**Conclusion:**

A new appearance model is introduced with several configurations presented and evaluated. The DAPs can be readily applied for other clinical problems for the tasks of prior learning, landmark detection and pathology classification.

## Introduction

**Fig. 1:**
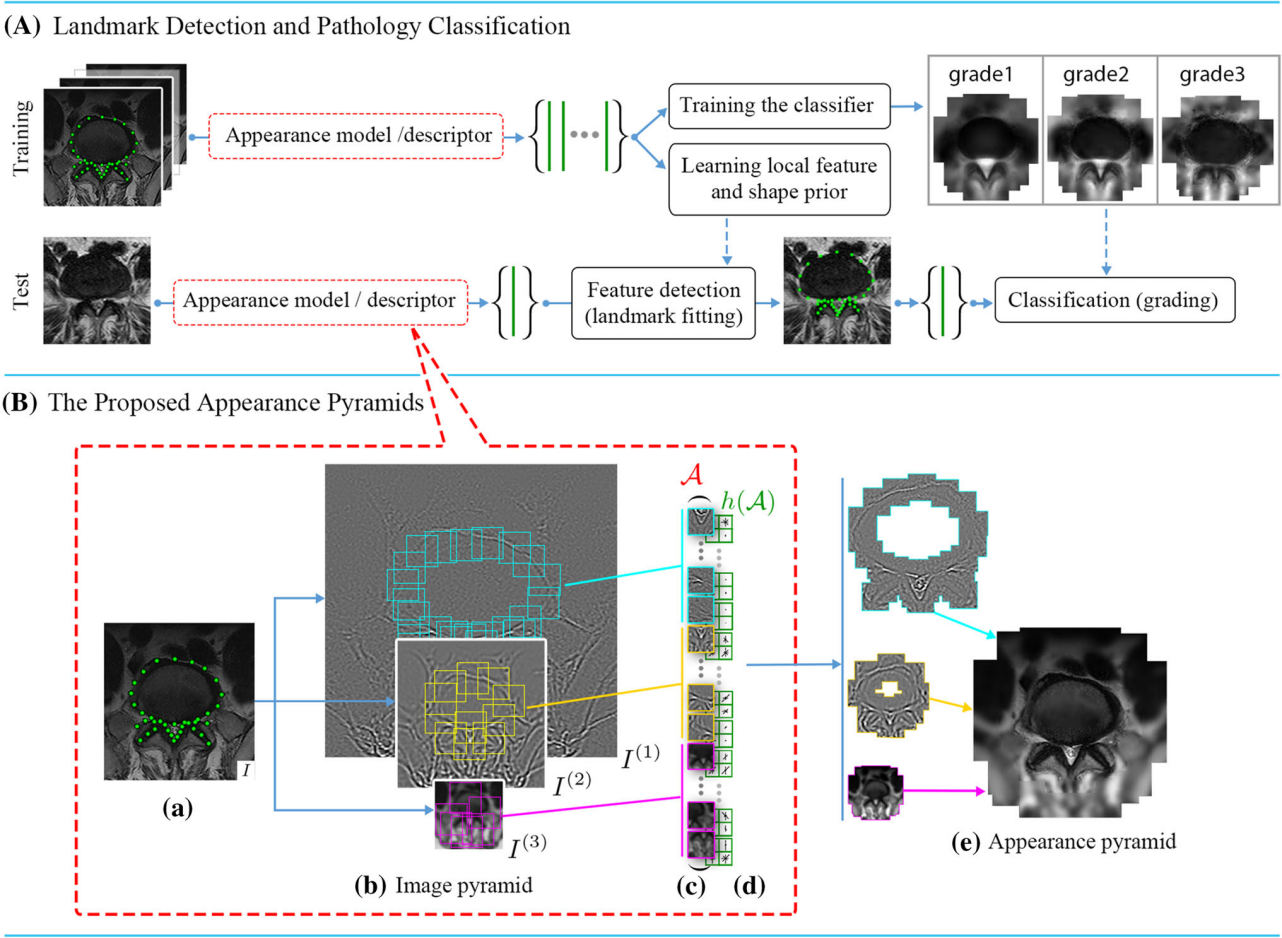
**a** A standard approach of landmark detection and pathology classification. **b** The proposed appearance model $\mathcal {A}$ and feature descriptor $h(\mathcal {A})$. *Appearance decomposition* (*a*) an image example. (*b*) Pyramidal image channels. Local patches are extracted from the channels at key landmarks in $\mathbf {s}$. Patches of different channels have the save size in pixels, which give a multi-scale description of the local features. (*c*) All patches are concatenated and flattened into a 1D vector $\mathcal {A}$ serving as the profile of the appearance. (*d*) A further feature extraction function can be used to enhance the robustness. *Reconstruction* (*e*) feature patches are padded at each scale level with the geometry configured by $\mathbf {s}$. All scales are accumulated to recover the object appearance

Object class representation is of vital importance for medical image analysis tasks such as localising anatomical features and classifying pathological conditions. Parametric representation of an object category allows the leveraging of the prior knowledge by learning the statistics of the parameters in the population. The representations are often vectorised and used as inputs for training a classifier (Fig. 1a). The training data usually consist of instances with landmarks annotated at consistent anatomical features. The appearance correspondence across the instances is built by aligning a deformable appearance (e.g. active appearance model (AAM) [3]) or extracting local features at the landmarks [1, 8, 16]. During testing, the landmarks are detected in new, unseen instances, and the features are extracted and sent to the classifier for pathology classification. For a robust landmark detection, a prior model of the object class is learnt by formulating the statistics of the parameters, and the searching is conducted under the regularisation of the prior model. The deformable model is either holistic [3], which consists of the shape and aligned appearance, or part based [1, 8, 11, 16], which represents an object by locally rigid parts with a shape capturing the spatial relationships among parts. In deformable part models (DPMs), the fitting process is implemented by local feature searching followed by a regularisation imposed through a prior model of the global shape. Various types of DPM instances have been proposed utilising advanced feature detection algorithms such as boosted regression [5], random forests [8], regularised mean-shift [11], and shape optimisation methods such as pictorial structures [1] and nonparametric models [16]. However, less attention has been paid to optimising the appearance representation and preserving the anatomical details in medical imaging.

In this paper, we introduce a new appearance model referred to as deformable appearance pyramids (DAPs). The object appearance is delineated by an appearance pyramid (AP), which is a multi-scale part-based representation built on the image pyramid, see Fig. 1b. The deformation is approximated by the translations of the parts as well as the linear appearance variations in the assembly of the parts. The multi-scale delineation preserves the details of the anatomical features at high resolution, while captures the background information at lower resolution. We present and evaluate the DAPs built on two types of image pyramids, namely Gaussian and wavelet pyramids, and introduce two methods to model the prior and fit to new instances, one explicitly using a multivariate Gaussian model and subspace Lucas–Kanade (LK) algorithm [2], another implicitly using supervised descent method (SDM) [16].

We apply the DAPs to the problem of lumbar spinal stenosis (LSS) for fitting the landmarks and grading the central and foraminal stenosis [7, 14]. The performances of the DAPs with various configurations are evaluated and compared with classic methods such as active shape models (ASMs), AAMs [3] and constrained local models (CLMs) [4]. Experimental results show that the DAPs built on wavelet image pyramids [18] and driven by the SDM give the best performance on both landmark detection and pathology classification.

## Deformable appearance pyramids for object representation

Objects belonging to the same class (e.g. same anatomy from different cases) often share similar appearances. The appearances can be represented by a deformable model, which is fitted to individual cases by changing the parameters of the model. With the deformable appearance model, the variations in the population caused by the diversity of individual cases or the pathological degenerations can be parametrised, learned and used as prior knowledge for robust fitting and classification. A DAP is a deformable model representing the anatomies by multi-scale rigid parts as well as the geometrical configuration. It models the variability within a population with local translations and linear appearance variations in the assembly of the parts.

**Fig. 2:**
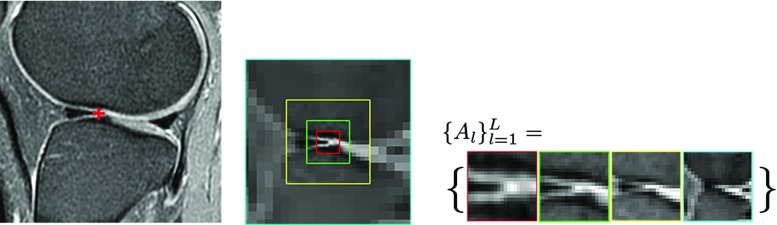
Gaussian local feature pyramid

### Local feature pyramid

We begin by describing the parts at multiple scales. The part at a landmark is typically described by an image patch with a certain size. Choice of the patch size can significantly affect the performance of the model. For sharper local structures, a smaller patch can give more precise pixel location. At blurry structures, however, the patch size should be large enough to cover distinguishable textural information. A good feature descriptor is expected to have a high spatial specificity (pixel location) while maintaining good distinctive ability (textural properties). Due to the uncertainty principle in signal processing [15], a single scale patch cannot achieve both. We therefore represent the part with a multi-scale local feature pyramid (LFP), with the smaller scales containing local high frequency features, and the larger scales low frequency components.

A *L*-level LFP at a landmark, denoted by $\{A_l\}_{l=1}^L$, is an assembly of patches extract from a *L*-level image pyramid. The patches $A_l$ describe the local features with increasing scales and decreasing resolutions in octave intervals. The first-level patch is the smallest one with the finest resolution. A patch in the *l*th level has *l* octaves larger scale and lower resolution, which keeps the same size in pixel across all levels, see an example extracted from Gaussian image pyramid in Fig. 2.

### Anatomy decomposition by DAP

A DAP is a part-based deformable model with each part delineated by a LFP. The DAP consists of two components:$\{\mathcal {A}, \mathbf {s}\}$, with $\mathcal {A}=\{\{A_{n,l}\}_{l=1}^L\}_{n=1}^N$, called an appearance pyramid, being the assembly of the LFPs, and $\mathbf {s}$ the geometrical configuration accounting for the deformations. *N* is the total number of landmarks.

As the patches cover larger anatomical regions at lower-resolution pyramidal layers, fewer number of patches are required to describe the appearance of the anatomy at a coarser level. We trim the patches at these levels preserving only those denoting key features. In practice, a simple trimming algorithm can be designed to iteratively delete the patches which have least distance from their neighbourhood patches until a distance criterion is satisfied. Denoting $\mathcal {K}_n$ as the subset of scale indices preserved at the *n*th landmark, the AP becomes $\mathcal {A} = \{\{A_{n,l}\}_{l\in \mathcal {K}_n}\}_{n=1}^N$. At each level of $\mathcal {A}$, the patches describe the anatomy with a certain degree of detail, and together, they give a multi-scale description, see Fig. 1b.

**Fig. 3:**
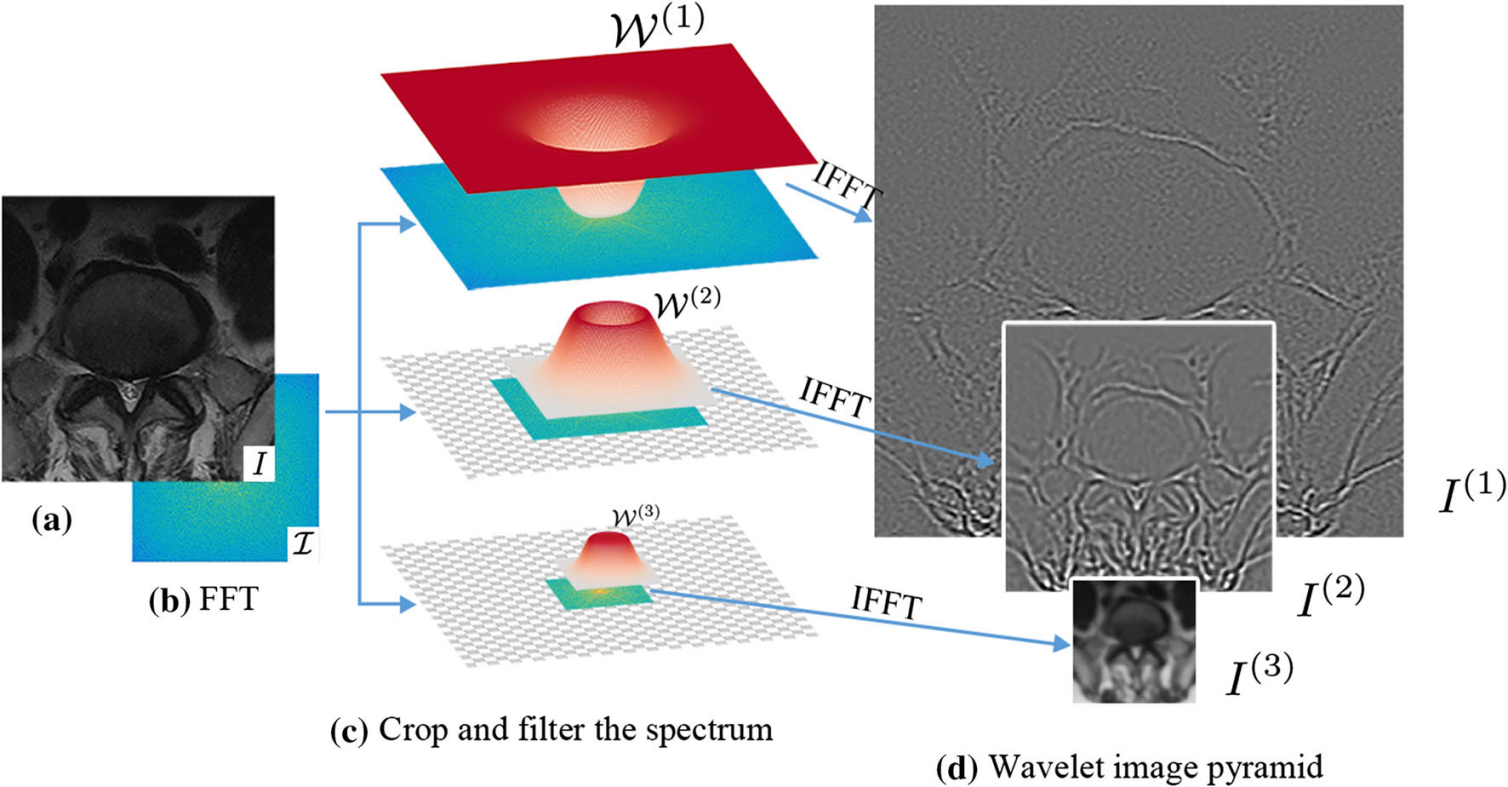
Wavelet image pyramid. (**a**) An image example. (**b**) Fourier transform of the image. (**c**) Multi-scale windows $\{\mathcal {W}^{(l)}\}_{l=1}^L$ are applied to the spectrum. As the windows cover only a subband at one octave lower, spectrums are cut by half at each larger scale. (**d**) Subband pyramids representing multi-scale structures are obtained directly from the filtered spectrum, with a simultaneous downsampling at larger scales achieved by the cropping in the Fourier domain

Various types of image pyramids can be used to build an AP for appearance delineation. To be able to preserve the full information of the anatomy and reconstruct the appearance, they are chosen to be either pyramids with redundant channels such as Gaussian pyramids or with complementary channels such as wavelet pyramids: we refer to the appearance delineations as Gaussian appearance pyramids and wavelet appearance pyramids, respectively. We briefly illustrate a recent method of wavelet pyramid decomposition in Fig. 3. A detailed introduction can be found in [18].^1^

## Deformable appearance pyramids fitting

Fitting a DAP to a new case is accomplished by searching for the landmarks based on local features and matching the model correctly to the geometry and appearance of the object. The geometrical configuration of a DAP defines how the parts relate to each other and the prior knowledge constrains the shape to be *plausible* in an object category. As a result, the choices of prior modelling and geometry constraint are important. We describe two strategies, one which learns the prior knowledge with explicit methods and the other implicitly.

### Explicit model

In the explicit method, the geometry is configured with the point distribution shape model. The shape is represented by $\mathbf {s} = [\mathbf {x}_1, \mathbf {x}_2,\ldots ,\mathbf {x}_N]$, in which $\mathbf {x}_n$ is the coordinate of the *n*th landmark. We follow the two-step fitting strategy commonly used in part-based models [11, 12], i.e. local feature searching followed by a geometrical regularisation. The local feature searching gives predictions of the landmark locations, while the shape prior regularises the geometry within plausible variations. The likelihood of a shape instance with respect to the shape prior and local landmark predictions can be calculated by,

1 $$\begin{aligned}&p(\mathbf {s}|{\varTheta },\mathcal {A}) \propto p(\mathbf {s}|{\varTheta }) p(\mathbf {s}|\mathcal {A}) \nonumber \\&\quad = p(\mathbf {s}|{\varTheta })\prod _{n=1}^N p(\mathbf {x}_n|\{A_{n,l}\}_{l\in \mathcal {K}_n}) \end{aligned}$$

We show how the prior of the patch appearance $\mathcal {A}$ is learnt and used for the local feature searching, and the prior of the geometry $\mathbf {s}$ is learnt for the shape regularisation.

#### Local feature searching

*Appearance prior* Given the training set, we can extract $\mathcal {A}$ from each image and obtain a set of training samples $\{\mathcal {A}_1, \mathcal {A}_2,\ldots \}$. By extracting the local features from the corresponding landmarks, the shape variation in the training set is removed and a better pixel-to-pixel correspondence achieved; therefore, $\mathcal {A}$ can be viewed as ‘shape-free’ appearances. To learn the statistics of the appearances, we normalise the mean and variance of each $\mathcal {A}$ and apply principal component analysis (PCA). The eigenvectors accounting for the significant variations in the training samples form a matrix $P_\mathcal {A}$, which spans an eigenspace.

A new instance can be represented in the eigenspace by

2 $$\begin{aligned} \mathcal {A} = \bar{\mathcal {A}} + P_{\mathcal {A}}\mathbf {b}_\mathcal {A}, \end{aligned}$$

in which $\bar{\mathcal {A}}$ is the average appearance and $\mathbf {b}_{\mathcal {A}}$ is the appearance parameters in the eigenspace, obtained by the projection,

3 $$\begin{aligned} \mathbf {b}_\mathcal {A} = P^\mathrm{T}_\mathcal {A}(\mathcal {A}-\bar{\mathcal {A}}). \end{aligned}$$

*Searching* We derive a subspace LK algorithm [2] for the DAP fitting. In a standard LK method, the searching can be expressed by

4 $$\begin{aligned} \hat{\mathbf {x}}_{n,l}=\arg \,\text {min}\,||A_{n,l}(\mathbf {x}_{n,l})-\bar{A}_{n,l}||^2, \end{aligned}$$

which attempts to find the location minimising the difference between the local appearance and the template $\bar{A}_{n,l}$. $A_{n,l}$ is the patch at the *i*th landmark and the *l*th scale in $\mathcal {A}$. $\bar{A}_{n,l}$ is a patch in $\bar{\mathcal {A}}$. ${\hat{\mathbf {x}}}_{n,l}$ is the predicted location of the *i*th landmark inferred from $A_{n,l}$.

The standard LK method assumes the difference between the template and the local feature is caused by the misalignment, and aims to minimise the difference by adjusting the location. However, the difference can also be the appearance variations among cases, which makes the searching challenging. As the salient variations have been learnt and represented in the eigenspace spanned by $P_\mathcal {A}$, we project the AP onto its orthogonal subspace where these variations are excluded, namely

5 $$\begin{aligned} \mathcal {A}^\bot = \left( I - P_\mathcal {A}P^\mathrm{T}_\mathcal {A}\right) \mathcal {A}, \end{aligned}$$

where *I* is an identity matrix. The objective function thus becomes

6 $$\begin{aligned} \hat{\mathbf {x}}_{n,l}=\arg \,\text {min}\,||A_{n,l}^\bot (\mathbf {x}_{n,l})-\bar{A}^\bot _{n,l}||^2, \end{aligned}$$

in which $A^\bot _{n,l}$ denotes a patch in $\mathcal {A}^\bot$. In this way, the salient appearance variations have been removed and a more robust LK method achieved. Equation (6) is solved iteratively by the inverse gradient descent method [17]

7 $$\begin{aligned} {\left\{ \begin{array}{ll} {\Delta }{\mathbf {x}}_{n,l} = \left( \frac{\partial \bar{A}^\bot _{n,l}}{\partial \mathbf {x}_n}\right) ^+ \left( A_{n,l}^\bot (\mathbf {x}_n)-\bar{A}_{n,l}^\bot \right) ,\\ \hat{\mathbf {x}}_{n,l} \leftarrow \hat{\mathbf {x}}_{n,l} + {\Delta }{\mathbf {x}}_{n,l} . \end{array}\right. } \end{aligned}$$

Suppose we also have the variance $\sigma ^2_{n,l}$ of the prediction $\hat{\mathbf {x}}_{n,l}$, which could indicate the salience of the local feature or the confidence of the prediction. To keep it simple, we calculate the variance as the mean squared difference between the patch observation and the template. Using a Gaussian parametric form, the likelihood of the location of the *i*th landmark given the multi-scale prediction can be represented by

8 $$\begin{aligned}&p(\mathbf {x}_n|\{A_{n,l}\}_{l\in \mathcal {K}_n}) \propto \prod _{l\in \mathcal {K}_n} p(\mathbf {x}_n|A_{n,l}) \nonumber \\&\quad =\prod _{l\in \mathcal {K}_n} \exp \frac{(\mathbf {x}_n-\hat{\mathbf {x}}_{n,l})^2}{-2\sigma _{n,l}^2}. \end{aligned}$$

#### Shape regularisation

*Shape prior* Assuming a multi-variant Gaussian model, the statistics of the shapes is built by applying PCA to the aligned training shapes,

9 $$\begin{aligned} \mathbf {b}_\mathrm{s} = P^\mathrm{T}_\mathrm{s}(\mathbf {s}-\bar{\mathbf {s}}), \end{aligned}$$

where $P_\mathrm{s}\in \mathbb {R} ^{2N\times t}$ is the eigenvectors matrix corresponding to the first *t* largest eigenvalues $\lambda _1,\ldots ,\lambda _t$ and spans a *t*-dimensional eigenspace. $\mathbf {b}_\mathrm{s}\in \mathbb {R}^{t\times 1}$ is the shape parameters in the eigenspace.

The probability of a shape instance being plausible in the eigenspace can be calculated by the density estimation [10],

10 $$\begin{aligned} p(\mathbf {s}|{\varTheta })\propto \exp \left( -\frac{1}{2}\mathbf {b}_\mathrm{s}^\mathrm{T}{\varLambda }\mathbf {b}_\mathrm{s}\right) =\exp \left( -\frac{1}{2}\sum _{j=1}^t\frac{b_j^2}{\lambda _j}\right) , \end{aligned}$$

in which $${\varLambda } = \text {diag}\{\lambda _1,\ldots ,\lambda _t\}$$.

*Regularisation* Substituting (8) and (10) into (1), the likelihood becomes

11 $$\begin{aligned}&p(\mathbf {s}|{\varTheta },\mathcal {A}) \propto \exp \left( -\frac{1}{2}\sum _{j=1}^t\frac{b_j^2}{\lambda _j}\right) \nonumber \\&\quad \prod _{n=1}^N \prod _{l\in \mathcal {K}_n} \exp \frac{(\mathbf {x}_n-\hat{\mathbf {x}}_{n,l})^2}{-2\sigma _{n,l}^2} \end{aligned}$$

Taking the negative log form, we can obtain an energy function,

12 $$\begin{aligned} E(\mathbf {s})= \frac{1}{2}\sum _{j=1}^t\frac{b_j^2}{\lambda _j} +\sum _{n=1}^N\sum _{l\in \mathcal {K}_n}\frac{(\mathbf {x}_n-\hat{\mathbf {x}}_{n,l})^2}{2\sigma ^2_{n,l}} \end{aligned}$$

The maximum likelihood shape with respect to the prior and observation is the one minimising $E(\mathbf {s})$, which is given by

13 $$\begin{aligned} \mathbf {s}= & {} \left( P_\mathrm{s}{\varLambda }^{-1}P_\mathrm{s}^\mathrm{T}+\sum _{l=1}^L{\varSigma }_l^{-1}\right) ^{-1} \nonumber \\&\quad \left( P_\mathrm{s}{\varLambda }^{-1}P_\mathrm{s}^\mathrm{T}\bar{\mathbf {s}}+\sum _{l=1}^L{\varSigma }^{-1}_l\hat{\mathbf {s}}_l\right) , \end{aligned}$$

where ${\varLambda }=\text {diag}([\lambda _1,\ldots ,\lambda _t])$ and ${\varSigma }_l=\text {diag}([\sigma ^2_{n,l},\ldots ,\sigma ^2_{N,l}])$. The detailed derivation 1 is given in “Appendix”.

### Implicit model

In the implicit model, we deduce the true shape $\mathbf {s}^*$ from the observation at an initial shape $\mathcal {A}(\mathbf {s}^{(0)})$, which is solving the regression problem, $\mathcal {A}(\mathbf {s}^{(0)})\mapsto \mathbf {s}^*$. With SDM algorithm, it can be decomposed into a set of regressors and fitted recursively,

14 $$\begin{aligned} {\left\{ \begin{array}{ll} \mathcal {A}(\mathbf {s}^{(i)})\mapsto {\Delta } \mathbf {s}^{(i)}, \\ \mathbf {s}^{(i+1)} = \mathbf {s}^{(i)}+{\Delta }\mathbf {s}^{(i)}. \end{array}\right. } \end{aligned}$$

Each regressor is modelled linearly by,

15 $$\begin{aligned} {\Delta }\mathbf {s}^{(i)}=R^{(i)}\mathcal {A}(\mathbf {s}^{(i)})+\mathbf {b}^{(i)}. \end{aligned}$$

The parameters $\{R^{(i)},\mathbf {b}^{(i)}\}$ can be learnt from the training images. Specifically, at the *i*th iteration, the parameters can be learnt by minimising the residual error of regression in the training set,

16 $$\begin{aligned} \mathop {\arg \,\text {min}}\limits _{\{R^{(i)},\mathbf {b}^{(i)}\}} \sum _{k=1}^M ||{\Delta } \mathbf {s}_k^{(i)}-R^{(i)}\mathcal {A}_k\left( \mathbf {s}^{(i)}_k\right) -\mathbf {b}^{(i)}||^2_2, \end{aligned}$$

in which *M* is the number of training samples. ${\Delta }\mathbf {s}_k^{(i)}$ is the difference between the current shape $\mathbf {s}^{(i)}$ and the true shape $\mathbf {s}^*_k$ of the *k*th training data. In all cases, the initial shape $\mathbf {s}^{(0)}$ for the first regressor is set as the average shape at the average location in the training dataset. The shape samples for training the subsequent regressors are generated by applying the previous regressor,

17 $$\begin{aligned} \mathbf {s}^{(i+1)}_k = \mathbf {s}^{(i)}_k+ R^{(i)}\mathcal {A}_k\left( \mathbf {s}_k^{(i)}\right) +\mathbf {b}^{(i)}. \end{aligned}$$

In practice, to suppress the over-fitting problem in these situations with high-dimensional features and inadequate training data, a L2 regularisation is applied and the objective function (16) becomes

18 $$\begin{aligned}&\mathop {\arg \,\text {min} }\limits _{\{ {R^{(i)}},{\mathrm{{b}}^{(i)}}\}} \sum _{k=1}^M ||{\Delta } \mathbf {s}_k^{(i)} \nonumber \\&\quad -R^{(i)}\mathcal {A}_k\left( \mathbf {s}^{(i)}_k\right) -\mathbf {b}^{(i)}||^2_2 +\lambda ||R^{(i)}||^2_2, \end{aligned}$$

where $\lambda$ controls the extent of regularisation. Note that in the implicit model the shape prior is in a nonparametric form and is integrated in the training of the regressors. More details of SDM can be found at Xiong and Torre [16].

To reduce the dimensionality of the descriptors and enhance the fitting performance, instead of using intensity features, a more robust feature descriptor such as histogram of oriented gradients (HOG) [6] can be readily applied on the patches. Denoting $h{(\cdot )}$ as the feature extraction function, the fitting process can be expressed by

19 $$\begin{aligned} {\left\{ \begin{array}{ll} {\Delta }\mathbf {s}^{(i)}=R^{(i)}h(\mathcal {A}(\mathbf {s}^{(i)}))+\mathbf {b}^{(i)}. \\ \mathbf {s}^{(i+1)} = \mathbf {s}^{(i)}+{\Delta }\mathbf {s}^{(i)}, \end{array}\right. } \end{aligned}$$

with the parameters $\{R^{(i)},\mathbf {b}^{(i)}\}$ learnt in the training data by

20 $$\begin{aligned}&\mathop {\arg \,\text {min}}\limits _{\{R^{(i)},\mathbf {b}^{(i)}\}} \sum _{k=1}^M ||{\Delta } \mathbf {s}_k^{(i)}-R^{(i)}h\left( \mathcal {A}_k\left( \mathbf {s}^{(i)}_k\right) \right) \nonumber \\&\quad -\mathbf {b}^{(i)}||^2_2 +\lambda ||R^{(i)}||^2_2. \end{aligned}$$

**Fig. 4:**
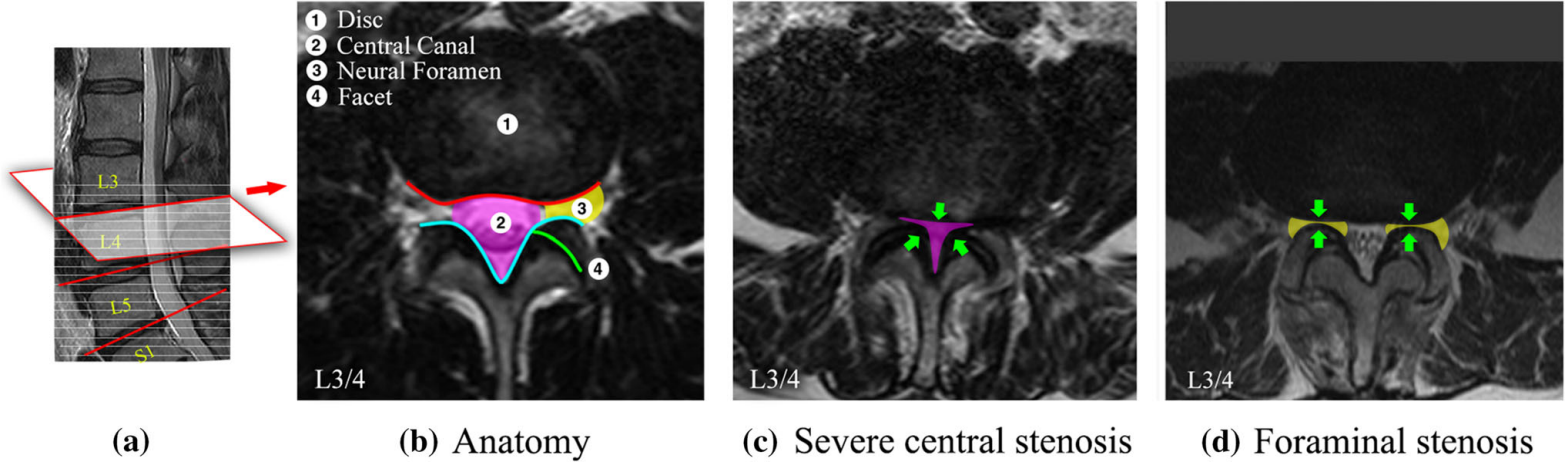
**a** Mid-sagittal view of a lumbar spine. *Grey dashed lines* show the raw axial scans. *Red lines* show the aligned disc-level planes, from which the axial images are extracted. **b** Anatomy of a L3/4 disc-level axial image. **c** A case with severe central stenosis. **d** A case with foraminal stenosis

**Table 1:** Performance of landmark detection by the criteria of PtoBD in pixels and DSC in percentage

Metrics	AAM	ASM	CLM	$\hbox {Gauss}+\hbox {LK}$*	$\hbox {Gauss}+\hbox {SDM}$*	$\hbox {Wavelets}+\hbox {SDM}$*
PtoBD	$3.10\pm 1.29$	$2.51\pm 1.32$	$2.34\pm 1.15$	$2.21\pm 1.07$	$1.95\pm 0.92$	1.87±0.73
DSC	$90.6\pm 4.9$	$92.1\pm 5.2$	$92.4\pm 5.2$	$92.8\pm 4.0$	$93.9\pm 3.3$	94.7±2.6

* Instances of DAP

### Appearance reconstruction, pathology modelling and classification

In the testing stage, the shape of an new object is fitted using the methods presented above. As the pyramidal channels are either redundant or complementary, we can recover the appearance of the object from the DAP. In other words, the objects can be represented compactly by the DAP parameters. Specifically, the shape parameters $\mathbf {b}_\mathrm{s}$ can be calculated by (9) and the appearance parameters $\mathbf {b}_\mathcal {A}$ by (3). For the classification tasks, the correspondence of anatomical features should be built such that the differences among the descriptors account for the true variations rather than the misalignment. In a DAP, the appearance correspondence is built by extracting local features at corresponding landmarks. A classifier predicts the label $\ell$ given an anatomical observation $\varPhi =[\mathbf {b}_\mathrm{s},\mathbf {b}_\mathcal {A}]$, i.e. $\ell = \arg \,\text {max} p(\ell | \varPhi )$. The most significant variations in the training data $\{\varPhi \}$ can be learned by a further PCA and the dimensionality reduced by preserving the significant components, which span a feature space $P_\varPhi$. A DAP therefore can be represented in the feature space by a compact set of parameters $\mathbf {b}_\varPhi$, i.e. $\mathbf {b}_\varPhi = P_\varPhi ^\mathrm{T} (\varPhi -\bar{\varPhi })$, in which $\bar{\varPhi }$ is the mean of $\{\varPhi \}$. Using $\mathbf {b}_\varPhi$ as inputs the classifier now predicts $\ell = \arg \,\text {max} p(\ell | \mathbf {b}_\varPhi )$. We train the classifier using AdaBoost with 100 learning cycles, with decision trees as the weak learners.

## Experiments

We apply the DAPs on the problem LSS for localising the feature landmarks and making pathological classification. LSS is a common disorder of the spine. The disorder can be observed in radiological studies as morphological abnormalities. Intervertebral disc-level axial images in MRI scans can provide rich information revealing the condition of important anatomies such as the disc, central canal, neural foramen and facet. In most cases, the original axial scans are not aligned to the disc planes caused by the curvature of the spine. To obtain the precise intervertebral views, we locate the disc planes in the sagittal scans (red line in Fig. 4) and map the geometry to the axial scans to calculate the coordinates, where the voxels are sampled to extract the aligned images. On a disc-level image shown in Fig. 4b, conditions of the posterior disc margins (red line) and the posterior spinal canal (cyan line) are typically inspected for the diagnosis. Degeneration of these structures can constrict the spinal canal (pink area) and the neural foramen (yellow area) causing central and foraminal stenosis.

The dataset for validation consists of T2-weighted MRI axial images of 200 patients with varied LSS symptoms. The L3/4, L4/5 and L5/S1 disc-level axial images are extracted, through which we obtain three sets of 200 axial images, 600 images in total. Due to the difference in resolution, all images are resampled to have a pixel space of 0.5 mm. Each image is inspected and labelled with respect to the conditions of central stenosis and foraminal stenosis, respectively. The anatomy is annotated with 37 landmarks outlining the disc, central canal and facet. We evaluate the performances of DAP with two choices of image appearances, i.e. Gaussian versus wavelets, and two choices of fitting methods, i.e. subspace LK versus SDM. We also compare them with three popular models: AAM [3, 9] as a standard appearance model, ASM as a widely used shape model, and CLM [4] as a part-based approach.

### Results of landmark detection

**Fig. 5:**
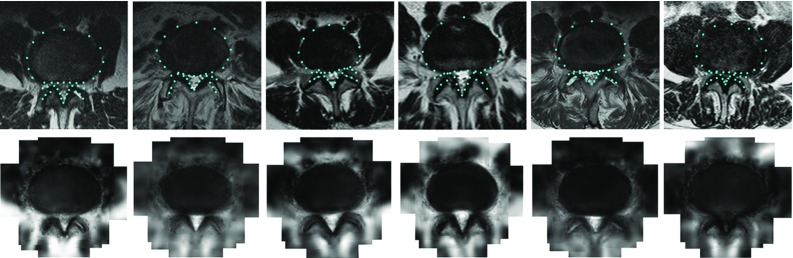
*Top* qualitative results of landmark detection by the DAP with wavelet appearance pyramid and SDM algorithm. *Bottom* appearance fitted by the wavelet DAP

For landmark detection, we evaluate the performance of DAPs with three configurations: Gaussian appearance pyramid with subspace LK as the fitting algorithm, Gaussian appearance pyramid with SDM and wavelet appearance pyramid with SDM. To cover richer pathological variations, we perform the landmark detection on the mixed dataset containing all 600 images. We randomly choose 300 images for training and detect the landmarks on the remaining 300. Two metrics are used for the evaluation: the point-to-boundary distance (PtoBD) and the dice similarity coefficients (DSC) of the canal and disc contours. PtoBD calculates the distance of the fitted landmarks to the ground truth contour, which is more accurate over point-to-point distance. DSC is defined as the amount of the intersection between a fitted shape and the ground truth, $\text {DSC}=2\cdot tp/(2\cdot tp+fp+fn)$, with *tp*, *fp* and *fn* denoting the true positive, false positive and false negative values, respectively. It considers both the sensitivity and specificity. The mean results of the methods compared are shown in Table 1. We can see that the DAPs with all three configurations outperform the other methods by a favourable margin. In addition, the comparison of the three DAP instances shows that the implicit model with SDM as the fitting algorithm gives better results than the explicit model with subspace LK as the fitting algorithm. Delineating the objects with wavelet appearance pyramids shows further improvement giving the best performance. Several qualitative results by the DAP with wavelet pyramids and SDM fitting algorithm are shown in Fig. 5.

**Fig. 6:**
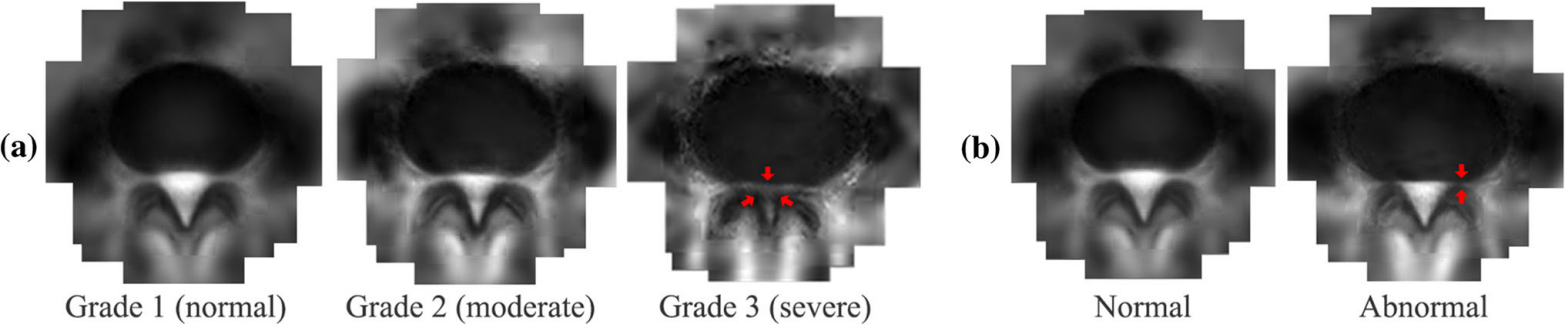
Average appearance of classes represented by wavelet DAP. **a** Three grades of central stenosis. **b** Normal and abnormal in terms of foreminal stenosis

**Table 2:** Agreement of classification and grading of central stenosis

Method	Accuracy (%) of classification			MAE of grading			RMSE of grading		
	L3/4	L4/5	L5/S1	L3/4	L4/5	L5/S1	L3/4	L4/5	L5/S1
ASM	$79.1\pm 4.8$	$77.4\pm 4.3$	$81.7\pm 4.5$	0.25	0.31	0.20	0.55	0.67	0.48
AAM	$70.1\pm 7.1$	$69.7\pm 7.3$	$71.3\pm 8.8$	0.41	0.44	0.32	0.72	0.79	0.58
CLM	$81.0\pm 4.9$	$82.4\pm 4.5$	$82.7\pm 4.4$	0.23	0.25	0.23	0.53	0.56	0.52
Gaussian DAP	$80.7\pm 4.9$	$82.1\pm 4.6$	$84.7\pm 4.2$	0.23	0.25	0.18	0.53	0.58	0.47
Wavelet DAP	$\mathbf{84.7} \pm {\mathbf{4.6}}$	$\mathbf{84.5}\pm {\mathbf{4.3}}$	${\mathbf{85.9}}\pm \mathbf{4.2}$	**0.19**	**0.21**	**0.16**	**0.48**	**0.54**	**0.44**

The best results are highlighted in bold

### Results of anatomical classification

After the landmarks are detected, the DAPs are extracted from the image and used as input for classification. As the SDM algorithm detects the landmarks with higher precision compared with a subspace LK method, we use the landmark locations by SDM in the classification tasks and evaluate the accuracy by Gaussian appearance pyramids and wavelet appearance pyramids.

**Table 3:** Accuracy ($\%$) of classification of foreminal stenosis

Anatomy	ASM	AAM	CLM	Gaussian DAP	Wavelet DAP
L3/4	$83.3\pm 3.8$	$73.3\pm 5.5$	$83.1\pm 4.7$	$84.3\pm 4.1$	$\mathbf{85.0}\pm \mathbf{3.9}$
L4/5	$82.4\pm 4.6$	$76.2\pm 5.8$	$83.3\pm 4.3$	$86.9\pm 3.9$	$\mathbf{87.8}\pm \mathbf{3.5}$
L5/S1	$81.8\pm 4.7$	$74.5\pm 5.7$	$82.9\pm 4.5$	$85.2\pm 4.3$	$\mathbf{85.7}\pm \mathbf{4.3}$

The best results are highlighted in bold

For central stenosis, in each of the three subsets, the morphology of the central canal is inspected and labelled with three grades: normal, moderate and severe. For illustration, the average appearances of these classes delineated by the wavelet DAP are shown in Fig. 6a. We randomly pick 100 samples to train the classifier and test on the remaining 100 and repeat for 100 times for an unbiased result. The DAP extracted from the detected landmarks are projected onto the feature space and represented by a compact set of parameters (Fig. 5, bottom), which are used as inputs of the classifier. The performance of normal/abnormal classification is measured by accuracy, which is calculated as $(tp+tn)/(tp+tn+fp+fn)$. The grading errors are measured with mean absolute errors (MAE) and root mean squared errors (RMSE). We compare the performance of DAPs against approaches using other models as inputs to the same classifier. The agreements of the results with manual inspection are reported in Table 2. We can see that the Gaussian DAP gives better or competitive performance in the classification and grading of the central stenosis, while the wavelet DAP outperforms the methods compared by a large margin. Similarly, we perform another normal/abnormal classification on the morphology of the neural foremen. The average appearances delineated by the wavelet DAP are given in Fig. 6b. The classification accuracy of methods compared is reported in Table 3. The result shows that the Gaussian DAP gives better performance compared with the popular shape and appearance models. The wavelet version of the DAP enables a further improvement. We believe that the DAP models benefit from its better local feature description and appearance delineation. The further improvement is brought on by the superior properties of wavelets, namely that they are complementary which preserves the full information of discriminating local appearance, and they decompose complex textures into simpler feature components.

## Conclusion

We presented a multi-scale deformable part model we refer to as a DAP. Several configurations of the DAP are introduced and evaluated, including two forms of pyramids, namely Gaussian pyramid and wavelet pyramid, and two fitting methods namely subspace LK and SDM. The models are applied on the problem of LSS for detecting the landmarks and classifying the pathologies. As the anatomies of cases at varied degree of degeneration are modelled and represented by the same compact parameters and the appearances can be reconstructed by the DAP models, suggested further work includes the combination of DAP and manifold learning methods such as anisotropic statistic modelling [13] to learn and visualise the pathological progress, by learning the most probable paths in the subspace. The DAPs can easily be applied to other anatomical area for clinical use where segmentation and classification are needed.

## Appendix: Derivation of the ML shape

The maximum likelihood shape is the one minimising the energy function,

21 $$\begin{aligned} E(\mathbf {s})=\sum _{j=1}^t\frac{b_j^2}{2\lambda _j}+\sum _{n=1}^N\sum _{l\in \ell }\frac{(\mathbf {x}_n-\hat{\mathbf {x}})^2}{2\sigma ^2_{n,l}}. \end{aligned}$$

We first rewrite it in a compact matrix form. To do so, we add to the equation a summation of zero terms,

22 $$\begin{aligned} \sum _{n=1}^N\sum _{l\in \ell }\frac{(\mathbf {x}_n-\hat{\mathbf {x}})^2}{2\sigma ^2_{n,l}}, \end{aligned}$$

with $\hat{\mathbf {x}}_{n,l}$ assigned with zero values and $\sigma _{n,l}^2$ set to be infinite. $\ell _n^C$ is the relative complement of $\ell _n$ in the number set $\{1,2,\ldots ,L\}$, indicating the missing levels at the *n*th landmark. The zero terms represent the estimations at landmarks of the trimmed patches in an DAP. The infinite variance value allows the landmark to lie anywhere.

With the zero terms, the energy function becomes

23 $$\begin{aligned} E(\mathbf {s})=\sum _{j=1}^t\frac{b_j^2}{2\lambda _j}+\sum _{n=1}^N\sum _{l=1}^L\frac{(\mathbf {x}_n-\hat{\mathbf {x}})^2}{2\sigma ^2_{n,l}}, \end{aligned}$$

which can be rewritten in a matrix form,

24 $$\begin{aligned} E(\mathbf {s})=\frac{1}{2}\mathbf {b}_\mathrm{s}^\mathrm{T}{\varLambda }^{-1}\mathbf {b}_\mathrm{s }+ \frac{1}{2}\sum _{l=1}^L(\mathbf {s}-\hat{\mathbf {s}}_l)^\mathrm{T}{\varSigma }_l^{-1}(\mathbf {s}-\hat{\mathbf {s}}_l), \end{aligned}$$

where ${\varLambda }=\text {diag}([\lambda _1,\ldots ,\lambda _t])$ and ${\varSigma }_l=\text {diag}([\sigma _{1,l}^2,\ldots ,\sigma ^2_{N,l}])$, $\mathbf {b}_\mathrm{s}$ is the vector of shape parameters and $\mathbf {s}$ is the shape.

Equation (24) has the typical form of an energy function for shape regularisation, with the difference that the second term is a summation of multiple predictions. Substituting 9 into 24 gives

25 $$\begin{aligned} E(\mathbf {s})=\frac{1}{2}(\mathbf {s}-\bar{\mathbf {s}})^\mathrm{T}P_\mathrm{s}{\varLambda }^{-1}P_\mathrm{s}^\mathrm{T}(\mathbf {s}-\bar{\mathbf {s}})+\frac{1}{2}\sum _{l=1}^L(\mathbf {s}-\hat{\mathbf {s}}_l). \end{aligned}$$

The ML shape $\mathbf {s}$ is the one minimising $E(\mathbf {s})$, obtained by solving the equation,

26 $$\begin{aligned} \frac{\text {d}E(\mathbf {s})}{\text {d}\mathbf {s}}=P_\mathrm{s}{\varLambda }^{-1}P_\mathrm{s}^\mathrm{T}(\mathbf {s}-\bar{\mathbf {s}})+\sum _{l=1}^L{\varSigma }_l^{-1}(\mathbf {s}-\hat{\mathbf {s}}_l) = 0. \end{aligned}$$

The solution is

27 $$\begin{aligned} \mathbf {s}= & {} \left( P_\mathrm{s}{\varLambda }^{-1}P_\mathrm{s}^\mathrm{T}+\sum _{l=1}^L{\varSigma }_l^{-1}\right) ^{-1} \nonumber \\&\quad \times \left( P_\mathrm{s}{\varLambda } P_\mathrm{s}^\mathrm{T}\bar{\mathbf {s}}+\sum _{l=1}^L{\varSigma }_l^{-1}\hat{\mathbf {s}}_l\right) . \end{aligned}$$

## Footnotes

1 Supplementary webpages of the Gaussian appearance pyramid can be found at http://sites.google.com/site/activeappearancepyramids/ and wavelet appearance pyramid at http://sites.google.com/site/waveletappearancepyramids/.
